# The impact of the COVID-19 pandemic on job satisfaction among professionally active nurses in five European countries

**DOI:** 10.3389/fpubh.2022.1006049

**Published:** 2022-09-28

**Authors:** Dawid Makowicz, Katarzyna Lisowicz, Krzysztof Bryniarski, Renata Dziubaszewska, Natalia Makowicz, Beata Dobrowolska

**Affiliations:** ^1^Department of Nursing, Carpathian State College, Krosno, Poland; ^2^Institute of Cardiology, Jagiellonian University, Kraków, Poland; ^3^Department of Holistic Care and Management in Nursing, Medical University, Lublin, Poland

**Keywords:** COVID-19, nursing, Europe, job satisfaction, pandemic

## Abstract

The COVID-19 pandemic has negatively affected the work of many medical professionals, including the group of nurses. This study aimed at assessing the impact of the COVID-19 pandemic on job satisfaction of nursing staff in five European countries. The study was conducted using the Job Satisfaction Scale (SSP) and original questions on the job satisfaction. The cross-sectional online study was conducted with a sample of 1,012 professionally active nurses working in Poland, Germany, Italy, Great Britain and Sweden, who assessed their job satisfaction before (retrospectively) and during the pandemic. The results showed a significant decrease in job satisfaction due to the need to perform it during the pandemic caused by the SARS-CoV-2 virus. In 8 out of 10 examined parameters of job satisfaction, a statistically significant decrease in job satisfaction was observed at the level of *p* < 0.05. Among the examined factors influencing job satisfaction, the highest decrease was recorded based on the assessment of working conditions (1,480). A high level of satisfaction with the work of nurses has a significant impact on providing better patient care as well as reducing the risk of professional burnout of nurses.

## Introduction

Humanity has struggled with different pandemics over the centuries. The most known, that have claimed the largest number of lives are plague, cholera and influenza (1). When a new type of pathogen belonging to already recognized group of coronaviruses was discovered in China, people were not aware of the effects of the new disease on the whole world could be. COVID-19 pandemic influenced human functioning globally: it reorganized professional work, people had to adapt to the prevailing restrictions and limit the possibility of contact with loved ones (2). The people who had to take care of patients infected with the SARS-CoV-2 virus from the early days of pandemic were nurses, whose work and personal life was completely changed. Due to the high risk of cross infection among the nursing staff, it was mandated that all professional activities and duties were to be performed using additional personal protective equipment, which included, although not limited to: filtering half masks, safety glasses, visors, overalls, medical gloves, and protective footwear or shoe protectors. Continuous work over several hours in such equipment within the dirty zone made it impossible to meet basic life needs (3). Fearing of the loved ones and to protect them for possible infection, some of the nursing staff decided to stay in hotels. Some were forced to temporarily leave their homes, due to being redeployed to work in single-named hospitals, even hundreds of kilometers away from their place of residence (4). Additional matter experienced by the medical staff was stigmatization, rejection and aggression from the surrounding society, often not only aimed at them directly, yet at their families too (4). The COVID-19 pandemic has caused enormous physical, as well as mental health burdens. It left its mark not only on the patients, but also on the people who cared for them. Nursing staff who encountered the disease and death of people infected with the SARS-CoV-2 virus on a daily basis often reported fear, mood disorders, anxiety, feelings of loneliness and sleep disorders (5–8). Research shows that lack of a negative test result for SARS-CoV-2 in a patient under care contributed to depression, anxiety and reduced professional job satisfaction (9). Nursing staff were concerned about their and their family's health. They were fully aware that they could be a potential source of infection for their family members (10). As the analyses reveal, the COVID-19 pandemic has significantly contributed to not only willingness, yet actual resignation among the nursing teams (11). Recent studies are limited to the respond from one specific country and therefore, it is difficult to compare the phenomenon of declining satisfaction among the nursing staff with their professional work in different countries, due to the use of different methodologies for conducting research (12–14). The decrease in job satisfaction can have a significant impact on the level of care provided to patients, as high-quality nursing care usually correlates with high level of job satisfaction. Low job satisfaction contributes to the omission of certain activities in patient care, which may cause adverse events and a general decline in the quality of services provided by nursing staff (15, 16). This survey is of an international nature, it aimed to compare the changes taking place in job satisfaction in different European countries during the COVID-19 pandemic among the nursing teams.

## Materials and methods

### Aim of the research

Aim of this research was to assess the impact of the COVID-19 pandemic on job satisfaction of nursing staff in five European countries.

### Research questions

1. In what aspects of nursing job satisfaction have been observed changes during the COVID-19 pandemic?
2. What determinants influenced the change in the level of job satisfaction of nursing staff during the COVID-19 pandemic?

### Study design

Cross sectional online study among 1,012 nurses from five European countries.

### Research instruments

The quantitative study was conducted using a survey consisting of 5 questions from the Job Satisfaction Scale (SSP) and 5 original questions; in all 10 queries, answers were given on a seven-point scale, where: 1 meant “I strongly disagree,” 4 “it is difficult to say whether I agree or disagree,” and 7 “I strongly agree” (17). Satisfaction of the professional work of male and female nurses before the COVID-19 pandemic and during the pandemic was assessed. The study assessed the average level of job satisfaction among nursing staff. Obtaining a higher level of answers indicated a higher level of satisfaction among the respondents. The scale was subjected to a reliability analysis (Alpha Cronbach), where the results were obtained-−0.915 (analysis before the COVID-19 pandemic) and 0.877 (analysis during the COVID-19 pandemic), which confirmed the reliability of the scale (18). The questions in the study also concerned the hospital wards where the respondents worked, the type of shift pattern performed, the average number of patients infected with the SARS-CoV-2 virus during one duty with which the respondents worked, the means of direct protection that they had, the opportunity to use in the ward during their work, and the possibility of taking a shower and disinfecting the body before leaving the ward following the completion of the shift. Additionally, respondents were asked about sociodemographic issues: country of origin, gender, age, place of residence and education. The survey was conducted in Polish for Polish-speaking respondents, for the remaining respondents, it was conducted in the English.

### Data collection

The material was collected from January to March 2022. The study was conducted using the Internet through the association of nurses' websites for nurses working in Poland, Germany, Great Britain, Sweden and Italy. The website selection criterion was to have at least 1,000 members. The members of the internet groups were actively working nursing staff in hospital departments of various specializations. Five websites were selected to survey all 5 countries and all have met above criterion. After the approval of the administrators of individual websites, a post was placed along with a survey and detailed information on the purpose and course of the study. The survey was completed by 1,067 respondents. From the collected material, questionnaires that were not completed and those in which inconsistencies appeared were removed. Eventually, the material in the form of 1,012 correctly completed surveys was subjected to analysis.

### Participants and setting

The study was addressed to nurses who, during the COVID-19 pandemic, worked or are working in the ward where patients infected with SARS-CoV-2 were treated or are being treated. The study 3 used the convenience sampling method. Each participant in the study had to meet the criterion of being a registered nurse and working regularly with the so-called COVID patient (for at least 30 days). It was assumed that the research will be conducted in 5 European countries located in different regions of Europe. Out of 46 European countries, Great Britain, Italy, Germany and Poland were selected due to the high incidence and mortality rates from COVID-19 (19),^1^ and Sweden, which, compared to other European countries, has opted for a policy of less stringent countermeasures, including mainly recommendations and guidelines instead of bans and orders (20). In each country, the number of nurses covered by the study was to oscillate around 200 respondents. Two hundred and fourteen (21%) nurses that were surveyed came from Poland, 208 (20.6%) from Great Britain, 202 (20%) from Germany, 196 (19.4%) from Italy, and 192 (19%) from Sweden.

### Ethical issues

At the beginning of the survey, information was provided about the aim of the study, the voluntary participation and the application of the obtained research results. Participants were informed that completing the online questionnaire will be considered and understood as giving consent to participate in the study. Respondents were also informed that they can withdraw at any point of the time during the data collection process. The survey was anonymous and did not violate the privacy of respondents. The study is in accordance with the requirements of the Declaration of Helsinki and the recommendations of the ICMJE. The Bioethics Committee agreed to carry them out (EC-0254/5/01/2022). In order to comply with the GDPR rules, the respondents consented to the processing of data included in the survey, the questions were designed to ensure that the respondents could not be identified. After data collection, the material was downloaded into an Excel file and stored on password protected computer.

### Statistical analysis

The basic test that was used in statistical analyses was the Chi-square test for the independence of variables. It was mainly used for questions built on nominal scales. To determine the strength of the compound, coefficients based on the aforementioned Chi-square and Kramer *V*-tests were used. The dependent variable was measured on a quantitative scale, and independent on a qualitative scale, while when the conditions for the use of parametric tests were not met, non-parametric tests for the assessment of differences in *U* Mann Whitney and Kruskal Wallis were used. Correlations between ordinal or quantitative variables (during unfulfilled conditions for the use of parametric tests) were made using Spearman's rho coefficient. Comparisons of dependent trials (before and after) were made using the *T*-test for dependent trials, which with equal groups is resistant to assumptions related to the use of parametric tests. The analysis was performed using the IBM SPSS 26.0 package together with the Exact Tests module—thorough tests. Any dependencies/correlations/differences are statistically significant with *p* ≤ 0.05.

## Results

### Characteristics of study participants and the conditions of professional work performed

The study group was dominated by women, constituting 89.7% of respondents (Table 1). The largest percentage of nurses (39%) was in the age range of 31–40 years. The mean age of nurses was 34 years (SD = 8.6). The majority of respondents lived in the city (70.4%) and 96.6% of respondents had a university degree. The place of work of the respondents was very diverse, the most people performed work in the infectious disease ward (11.7%). The respondents were dominated by people who worked in a two-shift system for 12 h (73.8%). The average number of patients infected with SARS-CoV-2 during one duty with which the nursing staff worked was 12 people, while the dominant number was 15 people.

**Table 1:** Sociodemographic data.

**Variable**	**Subgroup**	***N***	**%**
Sex	Woman	908	89.7%
	Man	104	10.3%
Age	21–30 years	383	37.9%
	31–40 years	395	39.0%
	41–50 years	167	16.5%
	Over 50 years	67	6.6%
Domicile	City	712	70.4%
	Village	300	29.6%
Education	Higher education	978	96.6%
	Secondary education	34	3.4%
Hospital ward	Department of infectious diseases	118	11.7%
	Pulmonology department	116	11.5%
	Intensive care unit	110	10.9%
	Internal medicine department	109	10.8%
	Rehabilitation department	91	8.9%
	Other departments	468	46.2%
Protection measures available	Medical gloves	1,012	100%
	Filtering half masks	905	89.4%
	Visors	851	84.1%
	Medical coverall	823	81.3%
	Shoe covers	762	75.3%
	Protective footwear	393	38.8%
	Safety glasses	378	37.4%
Possibility of bathing and disinfecting the body within the ward before leaving it	Yes	700	69.2%
	No	312	30.8%

The largest percentage of nurses working with COVID-19 patients had the opportunity to use medical gloves (100%) and filtering half masks (89.4%), while the smallest number of people had access to protective glasses (37.4%). At the end of duty (work), 69.2% of the respondents had the opportunity to bathe and disinfect their bodies within the ward before leaving (Table 1).

### Assessment of job satisfaction

#### Working conditions, the number of nursing staff to the number of patients, the social prestige of the profession

In most of the issues assessed (9/10), a decrease in professional work satisfaction was observed, while in 7/10 of the issues it was shown that the decrease was statistically significant at the level of *p* < 0.001 (Table 2). The greatest decrease in job satisfaction occurred due to the perception of the assessed working conditions (1.480) and the inappropriate ratio of nursing staff on duty to the number of patients on the ward (1.166). The only aspect assessed that increased during the pandemic was the sense of performing an important profession for the general public (0.379) (Table 2).

**Table 2:** Impact of the pandemic on professional job satisfaction among the nursing staff.

**Results for dependent variables**					**Differences in dependent variables**		***p***
**All countries**		**Average**	***N***	***SD***	**Average**	***SD***	
1	(Before COVID-19) In many ways, my work is close to perfect.	5.30	1,012	1.25	0.674	1.10	< 0.001
	(During COVID-19 course) In many ways, my work is close to perfect.	4.62	1,012	1.43			
2	(Before COVID-19) I have great working conditions	5.51	1,012	1.11	1.480	1.18	< 0.001
	(During COVID-19 course) I have great working conditions	4.03	1,012	1.45			
3	(Before COVID-19) I'm satisfied with the work	5.53	1,012	1.08	0.698	1.17	< 0.001
	(During COVID-19 course) I'm satisfied with the work	4.83	1,012	1.38			
4	(Before COVID-19) So far, at work, I have managed to achieve what I wanted	5.25	1,012	1.15	0.559	0.91	< 0.001
	(During COVID-19 course ) So far, at work, I have managed to achieve what I wanted	4.69	1,012	1.22			
5	(Before COVID-19) If I had to decide again, I would choose the same job	5.64	1,012	1.17	0.603	0.98	< 0.001
	(During COVID-19 course) If I had to decide again, I would choose the same job	5.04	1,012	1.21			
6	(Before COVID-19) I am satisfied with the remuneration I receive for my work	5.19	1,012	1.39	0.024	1.24	0.669
	(During COVID-19 course) I am satisfied with the remuneration I receive for my work	5.17	1,012	1.54			
7	(Before COVID-19) I have a very good relationship with my colleagues	5.80	1,012	1.08	0.103	0.92	0.012
	(During COVID-19 course) I have a very good relationship with my colleagues	5.70	1,012	1.05			
8	(Before COVID-19) I feel that my profession is important to the general public	5.39	1,012	1.12	−0.379	1.06	< 0.001
	(During COVID-19 course) I feel that my profession is important to the general public	5.76	1,012	0.97			
9	(Before COVID-19) My profession is associated with professional prestige	5.51	1,012	1.18	0.555	1.55	< 0.001
	(During COVID-19 course) My profession is associated with professional prestige	4.96	1,012	1.68			
10	(Before COVID-19) There is an adequate number of nursing staff on duty per patient	4.80	1,012	1.54	1.166	1.26	< 0.001
	(During COVID-19 course) There is an adequate number of nursing staff on duty per patient	3.63	1,012	1.62			

SD, Standard deviation. Fragments of tables marked in color show the statistical significance of specific results.

In Poland, among nurses, the greatest increase in the feeling that the profession is important to the general public was observed (0.897), while a significantly reduced response rate was noticed, compared to other countries indicating that the number of staff to the number of patients on a given ward is insufficient (0.206) (Table 3). This particular measure was also assessed in Poland prior pandemic and it was reported as much lower in contrast to other countries (3.06). In the United Kingdom, nurses were less likely to choose the nursing profession if there were given opportunity again, and it was noted as the highest level (1.192). Among the Italian respondents, the largest decrease in the overall assessment of working conditions was noted (2.959). The lowest decrease in satisfaction with the working conditions was observed by the Swedish respondents in contrast to the other countries surveyed (0.750). Interestingly, only Swedish data indicate an increase at the level of perceived professional prestige (0.104). In Germany, no significant deviations from the average scores were observed (Table 3).

**Table 3:** Impact of the pandemic on professional job satisfaction among the nursing staff in the surveyed European countries.

**Results for dependent variables**					**Differences in dependent variables**		***p***
**Poland**		**Average**	***N***	***SD***	**Average**	***SD***	
8	(Before COVID-19) I feel that my profession is important to the general public	4.63	214	1.04	−0.897	0.51	< 0.001
	(During COVID-19 pandemic) I feel that my profession is important to the general public	5.52	214	1.04			
10	(Before COVID-19) there is an adequate number of nursing staff on duty per patient	3.06	214	1.60	0.206	1.47	0.151
	(During COVID-19 pandemic) there is an adequate number of nursing staff on duty per patient	2.85	214	1.72			
**United Kingdom**							
5	(Before COVID-19) If I had to decide again, I would choose the same job	6.25	208	0.97	1.192	1.11	< 0.001
	(During COVID-19 pandemic) If I had to decide again, I would choose the same job	5.06	208	1.20			
**Italy**							
2	(Before COVID-19) I have great working conditions	5.46	196	0.78	2.959	1.03	< 0.001
	(During COVID-19 pandemic) I have great working conditions	2.50	196	0.73			
3	(Before COVID-19) I'm satisfied with the work	5.29	196	0.74	1.918	1.19	< 0.001
	(During COVID-19 pandemic) I'm satisfied with the work	3.37	196	0.89			
9	(Before COVID-19) my profession is associated with professional prestige	5.28	196	0.79	2.459	1.25	< 0.001
	(During COVID-19 pandemic) my profession is associated with professional prestige	2.82	196	0.93			
10	(Before COVID-19) there is an adequate number of nursing staff on duty per patient	3.92	196	0.68	2.194	1.05	< 0.001
	(During COVID-19 pandemic) there is an adequate number of nursing staff on duty per patient	1.72	196	0.84			
**Sweden**							
2	(Before COVID-19) I have great working conditions	5.63	192	0.63	0.750	0.82	< 0.001
	(During COVID-19 pandemic) I have great working conditions	4.88	192	0.93			
9	(Before COVID-19) my profession is associated with professional prestige	5.73	192	0.70	−0.104	0.96	0.294
	(During COVID-19 pandemic) my profession is associated with professional prestige	5.83	192	0.90			

SD, Standard deviation. Fragments of tables marked in color show the statistical significance of specific results.

#### Assessment of job satisfaction in individual countries

The conducted study showed that the highest decrease in job satisfaction by nursing staff was observed in Italy (1.0980), while the lowest decrease in satisfaction was recorded in Sweden (0.2958)—Table 4. In each of the countries, a statistically significant decrease (*p* < 0.05) was observed in each of the assessed job satisfaction factors (Table 4).

**Table 4:** The impact of the COVID-19 pandemic on job satisfaction of nursing staff in individual countries.

**Country by mortality rate**		**In many ways, my work is close to perfect. **	**I have great workingconditions**	**I'm satisfied with the work**	**So far, at work, I have managed to achieve what I wanted **	**If I had to decide again, I would choose the same job**	**I am satisfied with the remuneration I receive for my work **	**I have a very good relationship with my colleagues **	**I feel that my profession is important to the general public **	**My profession is associated with professional prestige **	**There is an adequate number of nursing staff on duty per patient **	**Satisfaction level **
Sweden	**Average**	–**0.4479**	–**0.7500**	–**0.2083**	–**0.4271**	–**0.3750**	–**0.3125**	–**0.0521**	**0.1563**	**0.1042**	–**0.6458**	–**0.2958**
	*N*	192	192	192	192	192	192	192	192	192	192	192
	*SD*	0.69	0.82	0.80	0.67	0.63	0.65	0.67	0.92	0.96	0.59	0.37
Poland	**Average**	–**0.6168**	–**1.3925**	–**0.5888**	–**0.3738**	–**0.2710**	–**0.4766**	–**0.0280**	**0.8972**	–**0.4299**	–**0.2056**	–**0.3486**
	*N*	214	214	214	214	214	214	214	214	214	214	214
	*SD*	1.04	0.90	0.87	0.68	0.92	1.64	0.60	0.51	1.54	1.47	0.61
Germany	**Average**	–**0.3465**	–**1.2277**	–**0.2376**	–**0.6436**	–**0.2673**	–**0.2574**	–**0.2970**	–**0.1386**	–**0.0990**	–**1.3564**	–**0.4871**
	*N*	202	202	202	202	202	202	202	202	202	202	202
	*SD*	0.72	0.82	0.61	0.86	0.83	0.84	0.62	0.88	0.93	0.79	0.40
United Kingdom	**Average**	–**0.5577**	–**1.0962**	–**0.5577**	–**0.6538**	–**1.1923**	**0.2692**	–**0.1538**	**0.0865**	**0.0577**	–**1.4808**	–**0.5279**
	*N*	208	208	208	208	208	208	208	208	208	208	208
	*SD*	1.27	0.95	1.34	1.11	1.11	1.26	1.45	1.20	1.30	1.14	0.82
Italy	**Average**	–**1.4184**	–**2.9592**	–**1.9184**	–**0.7041**	–**0.9082**	**0.6837**	**0.0204**	**0.8776**	–**2.4592**	–**2.1939**	–**1.0980**
	*N*	196	196	196	196	196	196	196	196	196	196	196
	*SD*	1.29	1.03	1.19	1.08	0.97	1.16	0.93	1.22	1.25	1.05	0.40
Total	Average	−0.6739	−1.4802	−0.6976	−0.5593	−0.6028	−0.0237	−0.1028	0.3794	−0.5553	−1.1660	−0.5482
	*N*	1,012	1,012	1,012	1,012	1,012	1,012	1,012	1,012	1,012	1,012	1,012
	*SD*	1.10	1.18	1.17	0.91	0.98	1.24	0.92	1.06	1.55	1.26	0.62
*H* Kruskal-Wallis		49.586	193.884	121.803	8.812	73.417	74.717	13.575	105.365	173.959	170.554	139.703
*P*		< 0001	< 0.001	< 0.001	< 0.001	< 0.001	< 0.001	0.009	< 0.001	< 0.001	< 0.001	< 0.001
*p* (Monte Carlo)		< 0.001	< 0.001	< 0.001	< 0.001	< 0.001	< 0.001	0.009	< 0.001	< 0.001	< 0.001	< 0.001

SD, Standard deviation. Fragments of tables marked in color show the statistical significance of specific results.

#### Assessment of the impact of gender on the perception of job satisfaction

Studies have shown the impact of gender on the job satisfaction during the COVID-19 pandemic in terms of perceiving the work as ideal (*p* = 0.005), assessment of working conditions (*p* < 0.001), satisfaction with previous achievements at work (*p* = 0.004) and assessment of nursing staff ration to patient (*p* = 0.015; Table 5). Globally, women in the study obtained a significant decrease in job satisfaction than men, the result was statistically significant (*p* = 0.011; Table 5).

**Table 5:** Change in satisfaction with professional work during the COVID-19 pandemic and the gender of the surveyed group.

**SEX**		**In many ways, my work is close to perfect.**	**I have great working conditions**	**I'm satisfied with the work**	**So far, at work, I have managed to achieve what I wanted**	**If I had to decide again, I would choose the same job**	**I am satisfied with the remuneration I receive for my work**	**I have a very good relationship with my colleagues**	**I feel that my profession is important to the general public**	**My profession is associated with professional prestige**	**There is an adequate number of nursing staff on duty per patient**	**Satisfaction level**
Woman	**Average**	–**0.7093**	–**1.5441**	**−0.7137**	**−0.5925**	**−0.6256**	**−0.0088**	**−0.0837**	**0.3767**	**−0.5815**	**−1.2004**	**−0.5683**
	*N*	908	908	908	908	908	908	908	908	908	908	908
	*SD*	1.12	1.20	1.21	0.92	1.00	1.27	0.95	1.07	1.57	1.27	0.63
Man	**Average**	**−0.3654**	**−0.9231**	**−0.5577**	**−0.2692**	**−0.4038**	**−0.1538**	**−0.2692**	**0.4038**	**−0.3269**	**−0.8654**	**−0.3731**
	*N*	104	104	104	104	104	104	104	104	104	104	104
	*SD*	0.84	0.78	0.72	0.74	0.82	1.01	0.48	1.01	1.27	1.17	0.53
Total	Average	−0.6739	−1.4802	−0.6976	−0.5593	−0.6028	−0.0237	−0.1028	0.3794	−0.5553	−1.1660	−0.5482
	*N*	1,012	1,012	1,012	1,012	1,012	1,012	1,012	1,012	1,012	1,012	1,012
	*SD*	1.10	1.18	1.17	0.91	0.98	1.24	0.92	1.06	1.55	1.26	0.62
*U* Mann-Whitney		9,163.5	8,395.5	11,296.5	9,138.5	10,295.0	11,076.5	10,118.0	11,691.0	10,993.0	9,469.5	9,281.5
*P*		0.005	< 0.001	0.594	0.004	0.107	0.447	0.057	0.905	0.403	0.015	0.011
*p* (Monte Carlo)		0.004	< 0.001	0.601	0.005	0.105	0.439	0.054	0.900	0.406	0.013	0.010

SD, Standard deviation. Fragments of tables marked in color show the statistical significance of specific results.

#### Assessment of the impact of age on the perception of job satisfaction

The decrease in the level of job satisfaction during the COVID-19 pandemic also depended on the age of respondents. The data were collected from assessing work as an ideal, job satisfaction, satisfaction with the professional goals achieved so far, re-choosing the same profession, satisfaction with pay, the importance of the profession for the general public and the assessment of professional prestige. In the 21–30 age range, there was a decrease at 0.4047, 31–40 years at 0.5614, 41–50 years at 0.6940, and over 50 years at 0.9333 (Table 6). As the age of the respondents increased, the overall level of satisfaction with their professional work decreased due to the emergence of the coronavirus pandemic (*p* < 0.001). During the pandemic, job satisfaction decrease was mostly reported among the oldest group of surveyed male and female nurses, and to the least extent in the youngest professional group (Table 6).

**Table 6:** Change in satisfaction with professional work during the COVID-19 pandemic and the age of the surveyed group.

**Age of male and female nurses**		**(In many ways, my work is close to perfect.)**	**I have great working conditions**	**I'm satisfied with the work**	**(So far, at work, I've been able to achieve what I wanted)**	**If I had to decide again, I would choose the same job**	**I am satisfied with the remuneration I receive for my work**	**I have a very good relationship with my colleagues**	**I feel that my profession is important to the general public**	**My profession is associated with professional prestige**	**There is an adequate number of nursing staff on duty per patient **	**Satisfaction level**
21–30 years	**Average**	**−0.4219**	**−1.3438**	**−0.5052**	**−0.4427**	**−0.5469**	**0.1510**	**−0.1094**	**0.5208**	**−0.3385**	**−1.0104**	**−0.4047**
	*N*	383	383	383	383	383	383	383	383	383	383	383
	*SD*	1.04	1.21	1.12	0.84	1.05	1.24	0.92	0.93	1.60	1.43	0.65
31–40 years	**Average**	**−0.7665**	**−1.5482**	**−0.7970**	**−0.6345**	**−0.5736**	**0.0660**	**0.0355**	**0.4975**	**−0.6294**	**−1.2640**	**−0.5614**
	*N*	395	395	395	395	395	395	395	395	395	395	395
	*SD*	1.05	1.18	1.19	0.90	0.88	1.12	0.83	0.94	1.55	1.13	0.48
41–50 years	**Average**	**−0.9167**	**−1.6190**	**−0.7143**	**−0.5238**	**−0.6071**	**−0.3929**	**−0.2619**	**0.0119**	**−0.7024**	**−1.2143**	**−0.6940**
	*N*	167	167	167	167	167	167	167	167	167	167	167
	*SD*	1.18	1.18	1.09	0.96	0.90	1.38	0.893	1.26	1.42	1.17	0.72
Over 50 years	**Average**	**−0.9697**	**−1.5152**	**−1.1818**	**−0.8788**	**−1.0909**	**−0.6364**	**−0.4848**	**−0.2121**	**−1.0000**	**−1.3636**	**−0.9333**
	*N*	67	67	67	67	67	67	67	67	67	67	67
	*SD*	1.31	0.87	1.44	1.08	1.28	1.27	1.27	1.53	1.41	1.08	0.73
Total	Average	−0.6739	−1.4802	−0.6976	−0.5593	−0.6028	−0.0237	−0.1028	0.3794	−0.5553	−1.1660	−0.5482
	*N*	1,012	1,012	1,012	1,012	1,012	1,012	1,012	1,012	1,012	1,012	1,012
	*SD*	1.10	1.18	1.17	0.91	0.98	1.24	0.92	1.06	1.55	1.26	0.62
*H* Kruskal-Wallis		18.425	7.187	10.068	9.025	9.050	15.284	5.505	18.124	9.969	5.127	24.861
*p*		< 0.001	0.066	0.018	0.029	0.029	0.002	0.138	< 0.001	0.019	0.163	< 0.001
*p* (Monte Carlo)		< 0.001	0.06	0.018	0.027	0.025	0.001	0.133	< 0.001	0.018	0.161	< 0.001

SD, Standard deviation. Fragments of tables marked in color show the statistical significance of specific results.

## Discussion

Satisfaction with the performed professional work is one of the most important factors that affect the efficiency of the work of medical staff, ensuring the highest possible core provided to the patient. In addition, it specifically prevents the phenomenon of burnout among health care workers (9). The current study showed a significant decrease in satisfaction with their professional work among nurses caused by work during the COVID-19 pandemic. The decrease in job satisfaction, in most of the surveyed aspects, was statistically significant. Said and El-Shafei conducted a study in which they found that nursing staff working in single-name wards during the pandemic showed significantly lower levels of job satisfaction than staff who worked in general wards and had no contact with COVID-19 patients (13). Similar analyses were presented by Savitsky et al. (21). The research conducted among physicians showed the adverse impact of the pandemic on job satisfaction, the authors also observed a large increase in burnout caused by the need to work during the pandemic (22). Sharif et al. proved that the excessive workload resulting from the current pandemic significantly reduces the perceived level of satisfaction within the medical profession (23). The researchers emphasize that the fear of getting ill and infecting the family with the SARS-CoV-2 virus has a significant impact on lowering the level of job satisfaction (24). The study abd-Ellatif et al. shows that 41.2% of respondents had a low level of job satisfaction due to fear of infection during the pandemic (25). According to the conducted research in a group of nurses working on wards where staff do not care for people suffering from COVID-19, 10% of respondents are seriously considering changing their profession, while on wards, where such patients are hospitalized, as many as 24.8% declare their willingness to change their occupation (13). In particular, De los Santos et al. and Labrague et al. underlined that the situation related to the need to work in new aggravating conditions led to very low job satisfaction among the nursing staff and effectively encouraged the decision to change professions (26, 27). The main reason for the decline in job satisfaction among nursing staff Soto-Rubio et al. report correlates to an increase in the prevalence of psychosocial risks during a pandemic, with a risk of accidents at work, low work commitment and mental illness (28). Low job satisfaction has a negative impact on the organizational commitment of health care workers, may contribute to staff shortages and is the main reason for the rotation of medical workers. Satisfied employees are more creative and dedicated to work, and engage in organizational tasks. Moreover, the conducted research showed a direct relationship between the satisfaction of health care workers and the satisfaction of patients with the care received during hospitalization (29). Employees who are more satisfied with their work, perform better in their workplaces and are more productive. The hospital management should take all efforts to ensure a high level of job satisfaction for their employees, as this will improve work efficiency, which consequently will provide better care for patients (30).

In the author's sample, the greatest decrease in professional satisfaction occurred due to the assessment of working conditions and the assessment of the ratio of nursing staff on duty to the number of patients on the ward. As shown by the research conducted by Havaei et al. there was a decrease in all aspects of working conditions surveyed among nursing staff, due to the emergence of the COVID-19 pandemic. At the same time, they underlined that the deterioration of working conditions prevented effective patient care (31). In addition, researchers noted a significant impact on deteriorating working conditions of the nurses' health (32). Lasater et al. (33) and Yu et al. (34) described an important problem regarding shortages of nursing staff, although highlighted it was already present before the pandemic, and during its duration, it simply became even more visible and problematic. An insufficient number of nursing staff on duty increases the risk of making mistakes when working with patients, which creates a risk to patients and the deterioration of their health. Higher patient mortality is observed in facilities where the ratio of nurses to patients was lower. In addition, the shortage of nursing staff increases the risk of dissatisfaction with the work performed and more frequent and faster burnout (35). Bad working conditions largely contribute to the resignation of nursing staff from work, and therefore, staff shortages and an insufficient number of nurses on duty are observed. The deteriorating working conditions prevent the nursing staff from providing the highest levels of care (36).

Changes in job satisfaction in individual countries were also analyzed. The lowest decline in job satisfaction was recorded in Sweden, in the United Kingdom the results were very close to the average of all countries, while the largest decrease was recorded in Italy. By analyzing mortality data in European countries (19) compared to the author's study, countries with higher mortality rates had a higher decline in job satisfaction. Aydin and Fidan proved that the incidence of patients' deaths during the SARS-CoV-2 pandemic on the ward where the nursing team had been working had a significant impact (*p* < 0.05) on their life satisfaction (37). Conducted research shows that patients' deaths are perceived by nursing staff as the most stressful factor in their professional work (38). Orrù et al. presenting their research, showed that medical staff who encountered the death of patients during the COVID-19 pandemic were much more likely to feel the negative effects of their work than staff who did not come into contact with the death of patients (39). On the other hand, Nwozichi et al. stressed that in addition to the deaths of patients, nursing staff often had to deal with the emotions and anxiety of deceased patients' loved ones, which further intensifies professional dissatisfaction (40). It would be extremely valuable to consider conducting a study involving the analysis of the relationship between job satisfaction and the direct number of deaths of patients experienced by nursing staff.

The author's research has shown that women, in most of the surveyed aspects (8/10), assessed their professional work as worse than men, due to the need to work during the COVID-19 pandemic. In 4, out of 10, parameters studied, the differences achieved were statistically significant, while globally the difference between the genders was presented at the level of statistical significance. Thai et al. also observed a significant difference between the satisfaction of women and men working in health care during the pandemic caused by the SARS-CoV-2 virus (41). Studies showing the multifaceted impact of the pandemic on the mental health of healthcare professionals revealed that in most of the parameters studied, the pandemic carried a much higher risk of complications for women: the risk of developing symptoms of depression, fear and anxiety. It should be emphasized that in no parameter studied did men report greater discomfort than women (42). The studies conducted among a group of physicians show that women are much more likely to show the negative impact of the current pandemic (22). De los Santos et al. proved that gender significantly affects the negative perception of professional work and leads to the fear of performing their profession (26). Other studies have also demonstrated that the SARS-CoV-2 pandemic has had a more negative impact on women working in healthcare than on men in each of the aspects studied (43–45). Ding et al. (46) who attempted to explain this phenomenon, noted that women were more sensitive and felt disgusted by the virus. In addition, they observed that the sex differences had already existed before and that the COVID-19 pandemic only made it more pronounced. Another factor that could have contributed to a higher decline in women's job satisfaction was a much greater increase in their domestic duties compared to men's, for example, due to the closure of childcare centers (47). The higher subjective perception of stressors in women may also be significant (48). The greater decline in satisfaction among women than in men is worrying because women constitute a much greater percentage of professionally active nursing personnel, which exacerbates the problem in this professional group (49). On the other hand, it is noteworthy that the study by Bettinsoli et al. rightly concluded that sharing their emotions and reporting mental health problems by men is considered less masculine. Therefore, there is a risk that men have also been less successful in dealing with the pandemic, however, they were not willing to expose their emotions and when answering the survey, they reported a lower decrease in job satisfaction than in reality (50).

The author's study showed that as the age of respondents increased, the level of satisfaction with their professional work decreased due to the emergence of the COVID-19 pandemic. In the oldest group of respondents, the decrease in job satisfaction was at the highest level, while in the youngest group the decrease in satisfaction was the lowest. This result is in line with results obtained by Majid et al. (51). The occurrence of this phenomenon has also been observed in groups of non-medical workers (52). The increased workload resulting from the pandemic is much more likely to increase the stress on older workers and their health. Senior nursing staff found it more difficult to adapt to the new epidemiological conditions, reducing their willingness to work (53). In the current epidemiological conditions, the pressure exerted on health care workers is constantly increasing. Compared to younger workers, middle-aged and older workers do not have as much capacity to relieve stress created in the workplace, due to family responsibilities and environmental factors (54).

## Limitations

The quantitative study provided information on the frequency of occurrence of a decrease in job satisfaction during the COVID-19 pandemic among the group of nurses. It seems reasonable to extend the research to other European countries along with a detailed qualitative analysis of the features that had a statistically significant impact on the decrease in job satisfaction. Additionally, when planning the extension of the study, consideration should planned to introduce additional factors that may significantly affect the satisfaction with the work of the nursing staff. A limitation of the study is the fact, that the respondents assessed their job satisfaction prior to the pandemic, retrospectively. Moreover, the respondents, countries of origin had different measures imposed during the pandemic and had different health care systems.

## Conclusions

Satisfaction with the work of nursing staff is extremely important to provide the highest possible level of care to patients. The COVID-19 pandemic has negatively affected the satisfaction of nurses with their professional work. It had the most severe impact on the perception of the change in working conditions. A greater decrease in job satisfaction was found among women and in the older group of nurses, in contrast to men and younger subjects. Demonstrating the existence of such a dependence should become an inspiration for the management in taking actions aimed at improving the current working conditions, which are considered insufficient by nurses during the pandemic.

## Data availability statement

The raw data supporting the conclusions of this article will be made available by the authors, without undue reservation.

## Ethics statement

The studies involving human participants were reviewed and approved by Bioethics Committee at the Medical University of Lublin. The patients/participants provided their written informed consent to participate in this study.

## Author contributions

DM, KL, KB, RD, NM, and BD contributed to conception and design of the study and wrote sections of the manuscript. DM, KL, and NM organized the database. DM performed the statistical analysis and wrote the first draft of the manuscript. All authors contributed to manuscript revision, read, and approved the submitted version.

## Funding

This work was financed from the funds of the Stanisław Pigon Scholarship of the Carpathian State College in Krosno.

## Conflict of interest

The authors declare that the research was conducted in the absence of any commercial or financial relationships that could be construed as a potential conflict of interest.

## Publisher's note

All claims expressed in this article are solely those of the authors and do not necessarily represent those of their affiliated organizations, or those of the publisher, the editors and the reviewers. Any product that may be evaluated in this article, or claim that may be made by its manufacturer, is not guaranteed or endorsed by the publisher.

## Footnotes

^1^https://www.worldometers.info/coronavirus/ (accessed January 15, 2022).

## References

[1] Krajewska H. Pandemie w historii świata. Wiet i Rolnictwo. (2020) 3:17–30. 10.53098/wir032020/01

[2] Hammerschmidt KSA Santana RF. Health of the older adults in times of the Covid-19 pandemic. Cogitare Enferm. (2020) 25:72849. 10.5380/ce.v25i0.72849

[3] Jackson D Bradbury-Jones C Baptiste D Gelling L Morin K Neville S. Life in the pandemic: some reflections on nursing in the context of COVID-19. J Clin Nurs. (2020) 29:2041–3. 10.1111/jocn.15257 32281185 PMC7228254

[4] Gniadek A Nawara W Padykuła M Malinowska-Lipień I. Polska pielegniarka w czasie pandemii zakazeń SARS-CoV-2 ARrózne perspektywy wykonywania zawodu. Zdrowie Publiczne i Zarziczneia. (2020) 18:149–54. 10.4467/20842627OZ.20.014.12767

[5] Szambor T Masiak J Urbańska A Składanowski M. Zwiazek pomiedzy sprawowaniem opieki medycznej nad osobami z choroba COVID-19, a zdrowiem psychicznym pracowników ochrony zdrowia. Pol J Public Health. (2019) 129:145–7. 10.2478/pjph-2019-0033

[6] Lai CC Shih TP Ko WC Tang HJ Hsueh PR. Severe acute respiratory syndrome coronavirus 2 (SARS-CoV-2) and coronavirus disease-2019 (COVID-19): the epidemic and the challenges. Int J Antimicrob Agents. (2020) 55:105924. 10.1016/j.ijantimicag.2020.105924 32081636 PMC7127800

[7] Ahn MH Shin Y Suh S Kim JH Kim HJ Lee K. High work-related stress and anxiety as a response to COVID-19 among health care workers in South Korea: cross-sectional online survey study. JMIR Public Health Surveill. (2021) 7:25489. 10.2196/25489 34478401 PMC8544732

[8] An Y Yang Y Wang A Li Y Zhang Q Cheung T. Prevalence of depression and its impact on quality of life among frontline nurses in emergency departments during the COVID-19 outbreak. J Affect Disord. (2020) 276:312–5. 10.1016/j.jad.2020.06.047 32871661 PMC7361044

[9] Zhang SX Liu J Jahanshahi AA Nawaser K Yousefi A Li J. At the height of the storm: healthcare staff's health conditions and job satisfaction and their associated predictors during the epidemic peak of COVID-19. Brain Behav Immun. (2020) 87:144–6. 10.1016/j.bbi.2020.05.010 32387345 PMC7199703

[10] Piredda M Fiorini J Marchetti A Mastroianni C Albanesi B Livigni L. The wounded healer: a phenomenological study on hospital nurses who contracted COVID-19. Front Public Health. (2022) 10:867826. 10.3389/fpubh.2022.867826 35875015 PMC9302606

[11] Ohue T Togo E Ohue Y Mitoku K. Mental health of nurses involved with COVID-19 patients in Japan, intention to resign, and influencing factors. Medicine. (2021) 100:26828. 10.1097/MD.0000000000026828 34397847 PMC8341249

[12] Pourteimour S Yaghmaei S Babamohamadi H. The relationship between mental workload and job performance among Iranian nurses providing care to COVID-19 patients: a cross-sectional study. J Nurs Manag. (2021) 29:1723–32. 10.1111/jonm.13305 33690932 PMC8236996

[13] Said RM El-Shafei DA. Occupational stress, job satisfaction, and intent to leave: nurses working on front lines during COVID-19 pandemic in Zagazig City, Egypt. ESPR. (2021) 28:8791–801. 10.1007/s11356-020-11235-8 33067794 PMC7567651

[14] Hoşgör H Yaman M. Investigation of the relationship between psychological resilience and job performance in Turkish nurses during the Covid-19 pandemic in terms of descriptive characteristics. J Nurs Manag. (2022) 30:44–52. 10.1111/jonm.13477 34595787 PMC8646929

[15] Mousazadeh S Yektatalab S Momennasab M Parvizy S. Job satisfaction challenges of nurses in the intensive care unit: a qualitative study. Risk Manag Healthc Policy. (2019) 12:233–42. 10.2147/RMHP.S218112 32009822 PMC6859118

[16] Plevová I Zeleníková R Jarošová D Janíková E. The relationship between nurse's job satisfaction and missed nursing care. Med Pr. (2021) 72:231–7. 10.13075/mp.5893.01035 33783436

[17] Lubrańska A Zawira E. Wolontariat a satysfakcja z zycia i satysfakcja z pracy. Edukacja Doroscjac. (2018) 1:121–32.

[18] Cizkowicz B. Omega McDonalda jako alternatywa dla alfa Cronbacha w szacowaniu rzetelności testu. Polskie Forum Psychologiczne. (2018) 23:311–29. 10.14656/PFP20180206

[19] Sanmarchi F Golinelli D Lenzi J Esposito F Capodici A Reno C. Exploring the gap between excess mortality and COVID-19 deaths in 67 countries. JAMA Netw Open. (2021) 4:2117359. 10.1001/jamanetworkopen.2021.17359 34269809 PMC8285734

[20] Askim J Bergström T. Between lockdown and calm down. Comparing the COVID-19 responses of Norway and Sweden. Local Gov Stud. (2022) 48:291–311. 10.1080/03003930.2021.1964477

[21] Savitsky B Radomislensky I Hendel T. Nurses' occupational satisfaction during Covid-19 pandemic. Appl Nurs Res. (2021) 59:151416. 10.1016/j.apnr.2021.151416 33947510 PMC7946538

[22] Alrawashdeh HM Al-Tammemi AB Alzawahreh MK Al-Tamimi A Elkholy M Al-Sarireh F. Occupational burnout and job satisfaction among physicians in times of COVID-19 crisis: a convergent parallel mixed-method study. BMC Public Health. (2021) 21:811. 10.1186/s12889-021-10897-4 33906619 PMC8079229

[23] Sharif H Arslan G Naghavi N Froelicher ES Kaveh O Sharif SP. A model of nurses' intention to care of patients with COVID-19: mediating roles of job satisfaction and organisational commitment. JCN. (2020) 30:1684–93. 10.1111/jocn.15723 33616249 PMC8014450

[24] Labrague LJ De los Santos JAA. Fear of COVID-19, psychological distress, work satisfaction and turnover intention among frontline nurses. J Nurs Manag. (2021) 29:395–403. 10.1111/jonm.13168 32985046 PMC7537256

[25] Abd-Ellatif EE Anwar MM AlJifri AA El Dalatony MM. Fear of COVID-19 and its impact on job satisfaction and turnover intention among egyptian physicians. Saf Health Work. (*2*021) 12:490–5. 10.1016/j.shaw.2021.07.007 34306797 PMC8284067

[26] De los Santos JAA Labragu LJ. The impact of fear of COVID-19 on job stress, and turnover intentionsof frontline nurses in the community: a cross-sectional study in the Philippines. Traumatology. (2021) 27:52–9. 10.1037/trm0000294

[27] Labrague LJ De Los Santos JAA. COVID-19 anxiety among frontline nurses: predictive role of organisational support, personal resilience and social support. J Nurs Manag. (2020) 28:1653–61. 10.1111/jonm.13121 32770780 PMC7436313

[28] Soto-Rubio A Giménez-Espert M del C Prado-Gascó V. Effect of emotional intelligence and psychosocial risks on burnout, job satisfaction, and nurses' health during the COVID-19 pandemic. Int J Environ Res Public Health. (2020) 17:7998. 10.3390/ijerph17217998 33143172 PMC7663663

[29] Akinwale OE George OJ. Work environment and job satisfaction among nurses in government tertiary hospitals in Nigeria. RAMJ. (2020) 14:71–92. 10.1108/RAMJ-01-2020-0002

[30] Karem MA Mahmood YN Jameel AS Ahmad AR. The effect of job satisfaction and organizational commitment on nurses' performance. HSSR. (2019) 7:332–9. 10.18510/hssr.2019.765835207009

[31] Havaei F Ma A Staempfli S MacPhee M. Nurses' workplace conditions impacting their mental health during COVID-19: a cross-sectional survey study. Healthcare. (2021) 9:84. 10.3390/healthcare9010084 33467080 PMC7830057

[32] Miranda FMA Santana LdeL Pizzolato AC Saquis LMM. Working conditions and the impact on the health of the nursing professionals in the context of covid-19. Cogitare Enferm. (2020) 25:72702. 10.5380/ce.v25i0.72702

[33] Lasater KB Aiken LH Sloane DM French R Martin B Reneau K. Chronic hospital nurse understaffing meets COVID-19: an observational study. BMJ Qual Saf. (2021) 30:639–47. 10.1136/bmjqs-2020-011512 32817399 PMC7443196

[34] Yu X Zhao Y Li Y Hu C Xu H Zhao X. Factors associated with job satisfaction of frontline medical staff fighting against COVID-19: a cross-sectional study in China. Front Public Health. (2020) 8:426. 10.3389/fpubh.2020.00426 32850610 PMC7417651

[35] Haddad LM Annamaraju P Toney-Butler TJ. Nursing Shortage. Treasure Island, FL: StatPearls (2022). 29630227

[36] Al Sabei SD Labrague LJ Ross AM Karkada S Albashayreh A Al Masroori F. Nursing work environment, turnover intention, job burnout, and quality of care: the moderating role of job satisfaction. J Nurs Scholarsh. (2020) 52:95–104. 10.1111/jnu.12528 31692251

[37] Aydin AK Fidan H. The effect of nurses' death anxiety on life satisfaction during the COVID-19 pandemic in Turkey. J Relig Health. (*2*022) 61:811–26. 10.1007/s10943-021-01357-9 34313909 PMC8313412

[38] Kwiecień-Jaguś K Medrzycka-Dabrowska W Chamienia A Kielaite V. Stress factors vs. job satisfaction among nursing staff in the Pomeranian Province (Poland) and the Vilnius Region (Lithuania). AAEM. (2018) 25:616–24. 10.26444/aaem/75801 30586980

[39] Orrù G Marzetti F Conversano C Vagheggini G Miccoli M Ciacchini R. Secondary traumatic stress and burnout in healthcare workers during COVID-19 outbreak. Int J Environ Res Public Health. (2021) 18:337. 10.3390/ijerph18010337 33466346 PMC7794988

[40] Nwozichi CU Guino TA Madu AM Hormazábal-Salgado R Jimoh MA Arungwa OT. The troubled nurse: a qualitative study of psychoemotional effects of cancer care on nurses in a Nigerian cancer care setting. Psychol Emot Effects Cancer Care. (2021) 18:328–35. 10.4103/apjon.apjon_25_20 33062827 PMC7529016

[41] Thai TT Le TAT Truong LTT Le NH Huynh ONH Nguyen TV. Care for the carers: an evaluation of job satisfaction of community healthcare workers in charge of infectious disease prevention and control in Vietnam. Risk Manag Healthc Policy. (2021) 14:2831–9. 10.2147/RMHP.S321314 34262370 PMC8274705

[42] Lai J Ma S Wang Y Cai Z Hu J Wei N. Factors associated with mental health outcomes among health care workers exposed to coronavirus disease 2019. JAMA Network Open. (2020) 3:3976. 10.1001/jamanetworkopen.2020.3976 32202646 PMC7090843

[43] Buselli R Corsi M Baldanzi S Chiumiento M Del Lupo E Dell'Oste V. Professional quality of life and mental health outcomes among health care workers exposed to SARS-CoV-2 (Covid-19). Int J Environ Res Public Health. (2020) 17:6180. 10.3390/ijerph17176180 32858810 PMC7504107

[44] Cui S Jiang Y Shi Q Zhang L Kong D Qian M. Impact of COVID-19 on anxiety, stress, and coping styles in nurses in emergency departments and fever clinics: a cross-sectional survey. Risk Manag Healthc Policy. (2021) 14:585–94. 10.2147/RMHP.S289782 33623449 PMC7894802

[45] Chatzittofis A Karanikola M Michailidou K Constantinidou A. Impact of the COVID-19 pandemic on the mental health of healthcare workers. Int J Environ Res Public Health. (2021) 18:1435. 10.3390/ijerph18041435 33546513 PMC7913751

[46] Ding Y Yang J Ji T Guo Y. Women suffered more emotional and life distress than men during the COVID-19 pandemic: the role of pathogen disgust sensitivity. Int J Environ Res Public Health. (2021) 18:8539. 10.3390/ijerph18168539 34444288 PMC8394728

[47] López-Atanes M Pijoán-Zubizarreta JI González-Briceño JP Leonés-Gil EM Recio-Barbero M González-Pinto A. Gender-based analysis of the psychological impact of the COVID-19 pandemic on healthcare workers in Spain. Front Psychiatry. (2021) 20:692215. 10.3389/fpsyt.2021.692215 34354613 PMC8329080

[48] Parmar K Solanki C Parikh M Vankar GK. Gender differences in stress at work place among doctors and nurses. GCSMC J Med Sci. (2015) 4:109–13.

[49] Martínez-Morato S Feijoo-Cid M Galbany-Estragués P Fernández-Cano MI Arreciado Marañón A. Emotion management and stereotypes about emotions among male nurses: a qualitative study. BMC Nurs. (2021) 20:114. 10.1186/s12912-021-00641-z 34182989 PMC8240313

[50] Bettinsoli ML Di Riso D Napier JL Moretti L Bettinsoli P Delmedico M. Mental health conditions of italian healthcare professionals during the COVID-19 disease outbreak. Appl Psychol Health Well Being. (2020) 12:1054–73. 10.1111/aphw.12239 33016564 PMC7675316

[51] Majid BHA Ebrahim T Narges K Zahra NK Farahnaz K Seyed Ehsan S. Relationships between job satisfaction and job demand, job control, social support, and depression in Iranian nurses. JNR. (2021) 29:2. 10.1097/jnr.0000000000000410 33156140

[52] John SF Varghese GM Varghese S. The impact of job satisfaction on job performance: an empirical analysis of virtual work during the pandemic. Palarch J Archaeol Egypt/Egyptol. (2020) 17:1503–10.

[53] Wu CH Lin HH Lai SY Tseng KC Hsu CH A. study of leisure constraints and job satisfaction of middle-aged and elderly health care workers in COVID-19 environment. Healthcare. (2021) 9:713. 10.3390/healthcare9060713 34200864 PMC8230547

[54] Idiegbeyan-Ose J Opeke R Aregbesola A Owolabi SE Eyiolorunshe T. Relationship between motivation and jobsatisfaction of staff in private university libraries, Nigeria. Acad Strat Manag J. (2019) 18:1–13.

